# The relationship between assessment methods and self-directed learning readiness in medical education

**DOI:** 10.5116/ijme.56bd.b282

**Published:** 2016-03-11

**Authors:** Katherine S. Monroe

**Affiliations:** 1 Emory University, School of Medicine in Atlanta, Georgia, USA

**Keywords:** Self-directed learning, assessment, evaluation

## Abstract

**Objectives:**

This research explored the assessment of self-directed learning readiness within the comprehensive evaluation of medical students’ knowledge and skills and the extent to which several variables predicted participants’ self-directed learning readiness prior to their graduation.

**Methods:**

Five metrics for evaluating medical students were considered in a multiple regression analysis.  Fourth-year medical students at a competitive US medical school received an informed consent and an online survey.  Participants voluntarily completed a self-directed learning readiness scale that assessed four subsets of self-directed learning readiness and consented to the release of their academic records.

**Results:**

The assortment of metrics considered in this study only vaguely captured students’ self-directedness.  The strongest predictors were faculty evaluations of students’ performance on clerkship rotations.  Specific clerkship grades were mildly predictive of three subscales.  The Pediatrics clerkship modestly predicted critical self-evaluation (r=-.30, p=.01) and the Psychiatry clerkship mildly predicted learning self-efficacy (r =-.30, p=.01), while the Junior Surgery clerkship nominally correlated with participants’ effective organization for learning (r=.21, p=.05).  Other metrics examined did not contribute to predicting participants’ readiness for self-directed learning.

**Conclusions:**

Given individual differences among participants for the variables considered, no combination of students’ grades and/or test scores overwhelmingly predicted their aptitude for self-directed learning.  Considering the importance of fostering medical students’ self-directed learning skills, schools need a reliable and pragmatic approach to measure them.  This data analysis, however, offered no clear-cut way of documenting students’ self-directed learning readiness based on the evaluation metrics included.

## Introduction

In 1950, the timeframe in which the body of medical knowledge doubles was estimated to be 50 years; in 2010, it was 3.5 years, and in 2020, it is projected to be just 73 days.^1^  The exponential growth of scientific knowledge is one of the most pervasive issues in medical education; to date, neither the expansion of curricula to fit the burgeoning scope nor the introduction of postgraduate and continuing medical education has accommodated it.^1^^-^^3^  Shojania and colleagues, in their 2007 study of 100 systematic reviews, showed that information gained through clinical research is relevant for approximately 5.5 years before a clinically pertinent change occurs.^4^  Most physicians, however, practice for an average of 30 years.^5^  Absorbing and incorporating information that changes at such an astounding rate presents a veritable challenge.  The concept of self-directed learning, which is an aspect of adult educational theory, has been offered as a way to address the need for doctors to maintain current information^6^^-^^9^ and essential in the “formation and ongoing competence of today’s physicians,”^10^  who will practice in an ever-changing world.

Self-directed learning was described as a distinct area of study in the 1960s and 1970s.^11^^,^^12^  Malcolm Knowles explicitly defined the concept as “a process in which individuals take the initiative, with or without the help of others, in diagnosing their own learning needs, formulating learning goals, identifying human and material sources for learning, choosing and implementing appropriate learning strategies, and evaluating learning outcomes.”^13^  Advocates assert that, by promoting a self-directed approach to learning, instructors (in the classroom or clinical environment) can serve as facilitators rather than lecturers, thus promoting a learner-centered approach to the educational process.^14^ Furthermore, scholars assert the importance of self-directed learning as a precursor to lifelong learning,^15^ and lifelong learning has been distinguished as one element of professionalism in medicine.^16^  Some characteristics of self-direction which are important for learning throughout life include a drive to learn independently, motivation to learn because of internal goals, a desire for personal growth, and the ability to steer further learning.^17^^,^^18^

Physicians must be able to recognize their deficiencies, search for current knowledge, and critically evaluate new research.^19^  The accrediting body of US medical schools, the Liaison Committee for Medical Education (LCME) has reinforced this sentiment; the Standards for Accreditation of Medical Education Programs Leading to the M.D. Degree published in March 2014 articulate the expectation that the “faculty of a medical school ensure that the medical curriculum includes self-directed learning experiences…to allow medical students to develop skills of lifelong learning.”^20^

Medical schools are increasingly challenged to cultivate professionals who have the knowledge and skills to be self-directed (lifelong) learners such that they are equipped to learn beyond their graduation and prepared to navigate a continually expanding body of knowledge.  Though the scholarly literature is replete with endorsements of adult learning principles and the promotion of self-directed learning in medical education, few studies have asserted their basic assertions,^9^  leaving a gap in the understanding of how self-directed learning readiness relates to medical students’ achievement. The LCME stipulates the possession of lifelong learning skills in graduates, yet little empirical evidence clarifies if self-directed learning readiness complements other metrics of success in medical schools or to what extent students are self-directed upon graduation.  Hence, the problem focus of this study was the increased challenge faced by medical schools to prepare physicians who have the knowledge and skills to be self-directed (lifelong) learners.  This research explored the relationship among various evaluation metrics of medical students at a US institution and their self-directed learning readiness (SDLR) to assess the extent to which they are related.

## Methods

This study utilized a quantitative methodology using multiple regression for statistical analysis.  Internal Review Board (IRB) approval was obtained at the institution prior to data collection.  Five independent variables (including cumulative grade point average [GPA], objective structured clinical evaluation [OSCE] scores, United States Medical Licensing Exam [USMLE] Step 1 and Step 2 Clinical Knowledge [CK], and clerkship evaluations) were considered, as well as descriptive and nuisance variables.  The independent (predictor) and potential nuisance variables were retrieved from archived institutional academic records that were obtained independent of this research as part of the comprehensive evaluation of medical students.  Participants’ scores on the Medical College Admissions Test (MCAT), a possible nuisance variable, were collected as part of their application to medical school (prior to their matriculation).  All other predictor variables were generated during participants’ advancement through an integrated medical curriculum and included both internal (within the institution) and external (national/standardized) evaluation metrics.  Cumulative GPAs reflect students’ overall performance in their didactic and clinical coursework, OSCE scores were measured during students’ second year, USMLE Step 1 scores were recorded in students’ second year, clerkship evaluations were conducted in students’ third year, and USMLE Step 2 CK scores were recorded in students’ fourth year.  All independent variables were collected (along with the dependent variables) by the researcher in participants’ final semester of medical school.  The dependent and descriptive (demographic) variables were self-reported by participants via survey responses.  Demographic variables were considered static and unaffected by medical school curriculum.

### Instrumentation

Hendry’s and Ginns’^21^ Self-Directed Learning Readiness Scale (SDLRS) was used to measure the four dependent variables in this study.  This instrument was specifically selected because it has been validated on medical students.  The scale consists of 36 items valued on a 5-point Likert scale ranging from ‘Strongly Disagree’ to ‘Strongly Agree.’  For the purpose of this research, each subscale of the SDLRS represented a dependent variable:  critical self-evaluation, learning self-efficacy, self-determination, and effective organization for learning.

### Research questions

To explore the relationship between various evaluation metrics of medical students and their self-directed learning readiness, four research questions were considered in this study.  Each question focused on a specific subset of Hendry’s and Ginns’ Self-Directed Learning Readiness Scale (SDLRS).  Accordingly, this study asked the following questions: 

  ·      **RQ1:**  Do evaluation metrics of medical students’ knowledge and skills predict their critical self-evaluation (subscale 1 of the SDLRS) during their fourth year of an integrated medical curriculum?

  ·      **RQ2:**  Do evaluation metrics of medical students’ knowledge and skills predict their learning self-efficacy (subscale 2 of the SDLRS) during their fourth year of an integrated medical curriculum?

  ·      **RQ3:**  Do evaluation metrics of medical students’ knowledge and skills predict their self-determination (subscale 3 of the SDLRS) during their fourth year of an integrated medical curriculum?

  ·      **RQ4:**  Do evaluation metrics of medical students’ knowledge and skills predict their effective organization for learning (subscale 4 of the SDLRS) during their fourth year of an integrated medical curriculum?

### Population, sample, and participants

The population for this study consisted of fourth-year students at a single institution who had progressed through an integrated medical curriculum. The Institution was purposefully selected as the site of study because of its curriculum design, which had recently been remodeled.  It is a competitive private south-eastern US medical school with a total student body of approximately 550 students.  Per the School of Medicine’s website, one intended goal of the new design is the graduation of “lifelong adult learners with the ability to take ownership of their own present and future educational needs.”^22^   Participants’ involvement in the study was voluntary.  All medical students who were on track to graduate in May 2014 were included in the sampling frame (N=124).  A total of 91 surveys were collected, and 14 were discarded during preliminary data screening because of errors, inconsistencies, and/or missing data, as recommended by Warner,^23^  yielding a final response rate of 61%. Participants self-reported their age, gender, and race/ethnicity, while the Registrar’s office provided their pre-admission MCAT scores.  Representativeness of the sample was verified by comparison of participants’ descriptive/demographic data against the comparable population data provided by the Institution, as recommended by Cook and colleagues.^24 ^ Table 1 summarizes the descriptive statistics of the sample and the population in this study.

**Table 1:** Descriptive statistics of sample and population

Variable		Sample	Population
Average age		27.3	26.6
Gender			
	Male	34 (44.2%)	59 (46.8%)
	Female	43 (55.8%)	67 (53.2%)
MCAT		33.5	33.5
Race/Ethnicity			
	American Indian/Alaska Native	1	0
	Asian	8	22
	Black/African American	8	10
	Hispanic/Latino/a	3	4
	White/Caucasian	54	76
	Other	2	14
	Missing/Incomplete	8	n/a

## Results

Descriptive statistics of the variables considered in this study are shown in Table 2.  Ultimately, one of the independent variables, participants’ OSCE score, was removed from the analysis because it was recorded by the Registrar as pass/fail, and passing the evaluation is a requirement for graduation.  Therefore, all participants received the same grade on the metric, and it provided no differentiation in the analysis.

The descriptive data suggest that, by and large, the participants in this research were strong students who performed well in courses and on exams.  This finding is not surprising, given that these individuals had successfully progressed through a competitive medical school.  Their responses on the Self-Directed Learning Readiness Scale suggest that they had an aptitude for self-directed learning.  Though no normative data is available from Hendry and Ginns’ validation study as a source of comparison, this finding is also not surprising; rather, it is congruent with the conclusions of McCune and colleagues,^25^Shokar and colleagues,^26^ and Findley and Bulik,^27^ who all reported a strong tendency for self-directed learning in the medical students they studied.

**Table 2:** Descriptive statistics of variables

Variable	N	Mean	Std. Deviation
Predictor			
Cumulative GPA	66	3.55	.26
USMLE Step 1	66	228.76	19.72
USMLE Step 2 CK	66	246.59	30.69
Clerkship Rotation			
MD 705	65	5.89 (B)	.99
MD 706	65	7.28 (A-)	.86
MD 710	65	6.35 (B+)	1.24
MD 715	62	7.40 (A-)	1.14
MD 720	65	6.12 (B+)	1.07
MD 725	65	7.02 (A-)	.82
MD 730	65	6.91 (A-)	1.03
MD 735	65	6.97 (A-)	1.40
MD 740	65	8.06 (A)	.90
MD 745	64	7.41 (A-)	1.20
Descriptive			
Age	77	27.27	2.32
Nuisance			
MCAT	66	33.53	2.51
Dependent			
Critical self-evaluation	77	4.08	.47
Learning self-efficacy	77	4.31	.28
Self-determination	77	3.68	.65
Effective organization for learning	77	3.68	.65

The strongest predictors of any component of medical students’ self-directed learning readiness were faculty evaluations of students’ performance on various clerkship rotations.  Two of the four SDLR subscales (critical self-evaluation and learning self-efficacy) were predicted at a statistically significant level (each by a different clerkship), and the Pearson correlations were moderate (r=-.30, p=.01) for each of those two subscales.  One of the subscales (effective organization for learning) nominally correlated with performance in a third clerkship (r=.21, p=.05).  Table 3 shows the correlations and significance of predictors for the subscales of the SDLRS.  Cumulative GPA, USMLE Step 1 score, and USMLE Step 2 CK score were not predictive of participants’ readiness to engage in self-directed learning.  Participants’ MCAT scores were not predictive of any subscale of the SDLRS, and the MCAT did not improve the predictive accuracy of any of the potential predictors. That being said, statistical theory suggests that the restricted range of MCAT scores in the sample (27-39 out of maximum score of 40) may have contributed to a lesser correlation than may have been seen in an unrestricted, normal distribution of scores.^28^

**Table 3:** Correlations and significance of predictors for SDLRS subscales

SDLRS Subscale	Predictive Independent Variable	*r*	*p*
1. Critical self-evaluation	MD725	-.30	.01
2. Learning self-efficacy	MD730	-.30	.01
3. Self-determination	none	n/a	n/a
4. Effective organization for learning	MD735	.21	.05

## Discussion

The findings of this research showed no strong individual differences in students’ self-directed learning readiness as a function of the independent variables considered.  Clerkship grades were mildly predictive of each of two subscales, critical self-evaluation (RQ1) and learning self-efficacy (RQ2), but they were inversely related:  participants who scored higher on the Self-Directed Learning Readiness Scale earned a lower grade in the predictive clerkship.  Though the correlation coefficient is small, this result is surprising and quite counter-intuitive. Perhaps it merely reflects sampling variance or undetected statistical artifact.  Alternatively, one must wonder if being strongly self-directed could actually distract a student from responsibilities on each of these rotations or if students’ self-directedness diverts their attention and effort away from meeting their supervisors’ goals.  None of the variables considered was predictive for the third subscale, self-determination. A third clerkship grade was slightly predictive of the final subscale, effective organization for learning (RQ4), with a positive Pearson correlation (r=.21, p=.05).  Students who scored highly on this subscale also did well in the clerkship, a result which contradicts the findings for the first two research questions.  The metrics selected in this study encompass a range of the methods by which medical students’ knowledge and skills are measured, but none of the evaluation metrics strongly captures students’ tendencies for self-directed learning, and the most statistically significant findings among the research questions contradict one another.  Though scholars assert the importance of self-directed learning, assessing it is ambiguous.

### Limitations

One limitation of this study is the potential difficulty in isolating variables related solely to self-directed learning in medical education.  Other possible explanations could contribute to an individual’s tendency for SDL.  For example, some studies^26^^,^^29^ have suggested that self-directed learners preferentially gravitate toward the medical field more than directed learners.  The medical school curriculum may also influence a student’s self-directed learning readiness. Whether students’ tendencies toward SDL increase as a result of a curriculum per se is also difficult to measure, and no school has ever scientifically documented the advantage of one curricular approach over another.^30^  This research did not attempt to control for these potential confounding variables, which are difficult to mitigate.  Rather, the purpose of this study was to explore the relationship between self-directed learning readiness and recognized metrics of competence in medical school.

Delimitations of the study include the medical school in which the study was conducted, the instrument used to collect data on self-directed learning readiness, and the evaluation metrics considered.  The institution was purposefully selected as the study site because of the curricular design that was intended to graduate “lifelong adult learners with the ability to take ownership of their own present and future educational needs.”^22^ Data regarding the self-directed learning readiness of students who matriculated prior to the curriculum remodel were not available, however, so this study is unable to compare results to previous cohorts.  The Self-Directed Learning Readiness Scale (SDLRS) was chosen in its revised form because of its documented validity with medical students.  Finally, the evaluation metrics were chosen because they are recognized assessment tools in the comprehensive evaluation of medical students. 

## Conclusions

The results of this study suggest more than one possible conclusion.  On the one hand, one could reasonably deduce that the participants were so similarly oriented toward self-directed learning that strong correlations between their learning styles and their grades did not emerge.  Perhaps the medical school encourages self-directed learning styles in all students, or maybe medical students enter their course of study with a high degree of self-directed learning readiness, and no intervention during the program affects it.  This study did not attempt to address this classic ‘nature vs. nurture’ debate.  Moreover, as Findley^29^ and Ludmerer^30^ assert, measuring the result of a curriculum can be quite difficult.  This study can only conclude that, given the individual differences among participants for the variables considered, no combination of students’ grades and/or test scores overwhelmingly predicted their aptitude for self-directed learning.

Alternatively, a second conclusion could be drawn that, despite the multiple examination methods conventionally used in medical education, the assortment considered in this study capture students’ tendencies for self-directedness only vaguely at best.  This analysis indicated that participants’ scores on three of the subscales of the Self-Directed Learning Readiness Scale were predicted by the grades they earned in each of three clerkship rotations.  Disconcertingly, however, two of these three subscales were inversely predicted - the most highly self-directed participants earned the lowest grades in the respective clerkships. Given the many ways in which medical students are evaluated during their progression through their medical curriculum, clerkship grades are arguably one of the most subjective assessments.  At the study site, clerkship grades are awarded as letter grades (with no numerical equivalent) based on the faculty’s estimation of the students’ academic and professional performance during their clinical rotations.  The learning environment, however, offers perhaps the least contrived setting.  Subject matter experts, with human judgment about how well people practice in real-world surroundings, appear to have the strongest sense of how self-directed their supervisees are, but they may fail to reward or reinforce highly self-directed behavior.

The negative correlations between clerkship grades and two subscales are perplexing.  Additionally, because a third subscale was positively predicted, it is difficult to postulate a common explanation.  During clerkship rotations, faculty members work with trainees in real-world clinical environments.  Though clerkship grades were perhaps the most subjective evaluation metric considered in this study, they do consider students’ approach to navigating their future work-a-day world.  Do faculty evaluations of students’ performance, as evidenced by their clerkship grades, provide reasonable data-driven documentation to clarify the extent to which medical students are self-directed upon graduation?  If so, are faculty members sufficiently equipped to make these judgments?  Holmboe and colleagues^31^ assert the importance of faculty development initiatives to offer formal training in assessment so that they are adequately prepared to “critically observe, question, and judge trainee performance” with patients and families in a real-world environment.  Would such initiatives strengthen the predictive capability of clerkship grades as a metric?

### Implications and recommendations for future research

Given that the evaluation techniques considered in this study did not provide a definitive assessment of students’ self-directed learning readiness, should there be another metric?  Should propensity for self-directed learning as an outcome be measured explicitly rather than interpreted from other metrics?  Continuing along this research path, it seems a logical question to ask:  in the course of medical education, is instilling an affinity for self-directed learning in medical students important enough that is should be empirically measured and documented specifically?  The literature indicates that fostering students’ self-directed learning skills should be a top priority for medical schools^32^^, ^^33^ thereby suggesting a need for a reliable and pragmatic approach to assessing them as an outcome.  The results of this study, however, suggest that either (1) the students who were studied are too similar for a statistical analysis to differentiate them based on the variables considered or (2) the conventional methods of assessing medical students that were considered in this study do not offer a clear-cut way to verify achievement of the outcome.

More can be learned about how self-directed learning readiness relates to other metrics of success in medical school.  This study was confined to one institution.  Data regarding the self-directed learning readiness of prior cohorts were not available, but the study could be repeated with future cohorts to see if findings would replicate.  The Self-Directed Learning Readiness Scale could be administered in the first year of the program as well as in the final semester prior to graduation to gauge changes in students’ self-directed learning readiness over the course of the curriculum.  Additionally, it would be interesting to see if, when repeated across a wider range of medical schools, the results would be similar.

## Acknowledgments

This study was conducted at Emory University School of Medicine in Atlanta, Georgia, United States of America.  The author declares no conflicts of interest.

## Conflict of Interest

The authors declare that they have no conflict of interest.
